# Structural studies of Helicase NS3 variants from Hepatitis C virus genotype 3 in virological sustained responder and non-responder patients

**DOI:** 10.1186/1756-0500-3-196

**Published:** 2010-07-14

**Authors:** Paola JS Provazzi, Helen A Arcuri, Isabel Maria VG de Carvalho-Mello, João Renato R Pinho, Maurício L Nogueira, Mário S Palma, Paula Rahal

**Affiliations:** 1 São Paulo State University - UNESP, Department of Biology, São José do Rio Preto/SP, CEP: 15054-000, Brazil; 2 Faculty of Medicine, University of São Paulo - USP, Department of Medical Clinic, São Paulo/SP, CEP: 01246-903, Brazil; 3 Butantan Institute, Viral Immunology Laboratory, São Paulo/SP, Brazil; 4 Faculty of Medicine, University of São Paulo - USP, Department of Gastroenterology, São Paulo/SP, CEP: 05503-900, Brazil; 5 Faculty of Medicine of São José do Rio Preto, São José do Rio Preto, CEP: 15090-000, Brazil; 6 São Paulo State University - UNESP, Center of Study of Social Insects/Department of Biology, Rio Claro/SP, CEP: 13506-900, Brazil

## Abstract

**Background:**

About 130 million people are infected with the hepatitis C virus (HCV) worldwide, but effective treatment options are not yet available. One of the most promising targets for antiviral therapy is nonstructural protein 3 (NS3). To identify possible changes in the structure of NS3 associated with virological sustained response or non-response of patients, a model was constructed for each helicase NS3 protein coding sequence. From this, the goal was to verify the interaction between helicases variants and their ligands.

**Findings:**

Evidence was found that the NS3 helicase portion of non-responder patients contained substitutions in its ATP and RNA binding sites. K210E substitution can cause an imbalance in the distribution of loads, leading to a decrease in the number of ligations between the essential amino acids required for the hydrolysis of ATP. W501R substitution causes an imbalance in the distribution of loads, leading and forcing the RNA to interact with the amino acid Thr269, but not preventing binding of ribavirin inhibitor.

**Conclusions:**

Useful information is provided on the genetic profiling of the HCV genotype 3, specifically the coding region of the NS3 protein, improving our understanding of the viral genome and the regions of its protein catalytic site.

## Background

Hepatitis C virus (HCV) is the main causative agent of non-A and non-B hepatitis. The clinical manifestations of infection include acute and chronic forms of hepatitis C, liver cirrhosis and hepatocellular carcinoma [1]. The overall prevalence estimated of HCV infection is 2.2%, which corresponds to 130 million HCV-positive people in the world [2]. About 3 million people in Brazil are infected [3].

The HCV genome contains a positive single-stranded RNA of ~9.6 kb. It encodes a single precursor polyprotein containing ~3000 amino acids [4-6], which gives rise to all viral structural proteins (S) - core (protein C), envelope 1 (E1) and envelope 2 (E2) - and nonstructural proteins (NS), located in the following order: NS2, NS3, NS4A, NS4B, NS5A, and NS5B [7].

The NS3 protein of the hepatitis C virus (HCV) is a target for development of antiviral agents. It is a hydrophobic protein of ~69 kDa, with its serine-protease function encoded in its N-terminal portion accounting for one-third of the entire protein [7]. The C-terminal portion of the structural protein NS3 corresponds to the helicase domain, having NTPase and RNA helicase activities [8].

To identify possible changes in the structure of the NS3 protein associated with virologically sustained responder and non-responder patients, a model was constructed for each helicase NS3 protein coding sequence.

## Methods

### Population and samples

The study material consisted of the serum samples of the 16 patients infected with hepatitis C virus genotype 3. After confirming the positive diagnosis of infection, defined by positivity for the virus antibody by ELISA and qualitative PCR for RNA, the patients were given 24 weeks of treatment with interferon-alpha and ribavirin, and were followed-up for up to 6 months after medication. Serum collections were made at 12 and 24 weeks during treatment, and 7, 14, 21 and 28 days after the treatment had been completed, then monthly for up to 6 months. Co-infection with the human immunodeficiency virus (HIV) and/or with the hepatitis B virus (HBV) was taken as an exclusion criterion. The project was approved by the research ethics committee of the São José do Rio Preto School of Medicine (FAMERP; opinion Nr. 087/2004).

### Molecular Modeling

For the modeling of NS3, the restrain-based modeling approach was used as implemented in the MODELLER program [9]. A total of 1000 models were generated for each clone and the final model was selected based on objective function and stereochemical quality.

The stereochemical evaluation quality of the final model was assessed by PROCHECK [10], X-PLOR [11], Verify-3D [12] and WHATCHECK [13] programs.

### Molecular Docking Simulations

The Molegro Virtual Docker program (version 2.0) was used to generate an ensemble of docked conformations for each variant helicase protein and ATP, RNA and Ribavirin compounds [14].

## Results and discussion

### Patients' characteristics

A total of 16 patients were treated and followed until 6 months after the end of the treatment. Seven patients (43.7%) presented a virological sustained response (VSR), of whom 6 were male and 1 was female. Nine patients (56.3%) presented non-response (NR), 6 being male and 3 female. Thirteen patients were infected with HCV genotype 3a and 3 with HCV genotype 3e. The patients' average age was 47.7 years (Table 1).

**Table 1:** Patients' characteristics

	SEX	AGE (YEARS)	HCV GENOTYPE	SUBSTITUTION	RESPONSE
**RF006**	M	39	3a		Non-responder
**RF007**	F	28	3e	K210E ATP binding site	Non-responder
**RF009**	M	51	3a		Non-responder
**RF015**	M	50	3a		Virological sustained responder
**RF018**	M	49	3a		Virological sustained responder
**RF020**	M	41	3e	W501R RNA binding site	Non-responder
**RF059**	F	54	3a	F444S	Virological sustained responder
**RF060**	M	48	3a		Non-responder
**RF061**	M	38	3e		Virological sustained responder
**RF075**	F	46	3a		Non-responder
**RF080**	M	52	3a		Virological sustained responder
**RF081**	M	58	3a		Virological sustained responder
**RF082**	F	57	3a		Non-responder
**RF096**	M	60	3a		Virological sustained responder
**RF115**	M	41	3a		Non-responder
**RF145**	M	51	3a		Non-responder

### Analysis of the substitutions in the sequences NS3 Helicase

Multiple sequence alignment analysis of the helicase fragment obtained from non-responder patients showed significant changes in helicase residues, for instance in the ATP and RNA binding sites. In patient RF007, a substitution of Lys210 for Glu210 occurred at the ATP binding site (Table 1). In the RNA binding site, patient RF020 had a Trp501 to Arg501 substitution (Table 1). No substitutions were found in the terminal region of the loop corresponding to a hydrophobic region (Phe531, Phe536 and Trp532). The arginine residue at position 393, important in the design of specific HCV NS3 inhibitors, was also assessed, but no changes were detected (Figure S1 of additional file 1).

PROTEUS showed variation in the number of α-helix, beta-sheet and coil structures in all resistant patients (diagram I of additional file 1). The PROTPARAM analysis showed the substitutions in the protein's amino acids composition which leads to and compromises the structure as a whole in resistant patients (diagram II of additional file 1). However, since these are theoretical models, experimental techniques will be used to confirm the structural differences observed in the helicase portion, and to investigate whether there is a reduction of its affinity for the ligands. The elements of the tertiary structure showing that the substitutions subtly alter the quantitation of the secondary structure in Non-Responder patients due an increase in the random structure regions are shown in Figures S2A-S2C of additional file 1.

### Analysis of the helicase ATP-binding site

**Figure 1:**
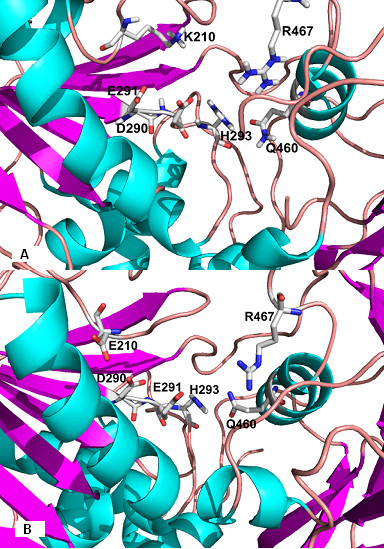
**Key residues involved in ATP binding: (A) Template (PDB**1A1V**; Kim et al., 1998) **[18]**, (B) patient RF007 showing the substitution of lysine for glutamic acid at position 210 of NS3**.

Mutations in motifs I to VI located in the helicase domains 1 and 2 can impact on the ability of the protein to unwind genetic material and hydrolyze ATP, showing that these two activities are co-operative. Here, we have highlighted the K210E substitution (Table 1), which leads to a change of a positive to a negative charge in the altered residue (Figures 1A and 1B), a different charge distribution and interactions between amino acids that might interfere with the hydrolysis of ATP.

**Figure 2:**
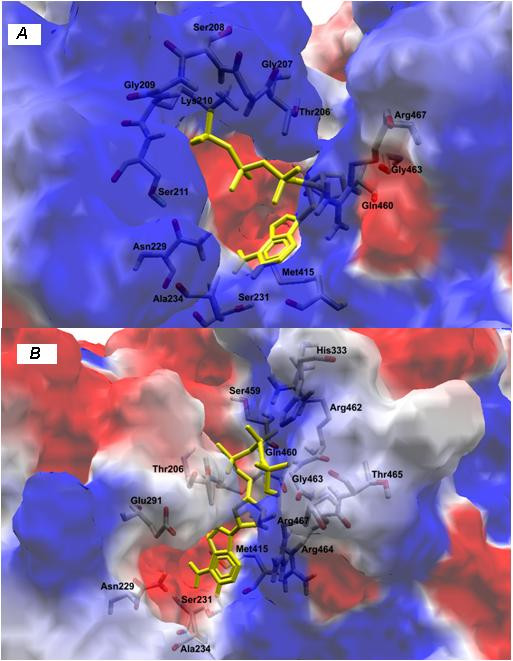
**Docking simulations between helicase and ATP: (A) patient RF059 and (B) patient RF007**.

To verify that the consequences the K210E substitution led to interaction with ATP, docking simulations were performed between helicase variant and the compost. The exchange of charges due to the substitution produced an imbalance in the distribution of loads, leading to a decrease in the number of ligations between the essential amino acids for the exercise of hydrolysis of ATP (Figures 2A-2B). ATP and a required metal ion cofactor (*i.e*. Mg2^+^) normally bind to a helicase in the cleft that separates two adjacent RecA-like domains. The most critical residues for ATP binding arise from the Walker A and B motifs. The Walker A motif of HCV helicase forms a phosphate binding loop (P-loop) with the conserved Lys210, thereby probably contacting the γ phosphate of ATP [15]. With this disturbance, substitution of Lys210 caused a mild disruption of the molecule accompanied by a change in its orientation.

Kim et al. [16] through site-directed mutations showed that the K210E mutant lost its RNA helicase activity and had very little NTPase activity compared to the wild-type protein. Similar results were observed by Chang et al. [17], in which the ATPase activity was significantly reduced in mutant NS3 proteins (K210E). They also observed that the wild type NS3 protein completely unwound the RNA substrate in the presence of ATP, but the mutation (K210E) completely abrogated the activity.

### Analysis of the helicase RNA-binding site

**Figure 3:**
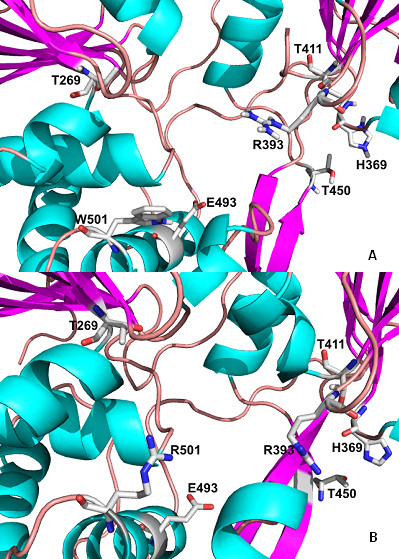
**(A) Key residues in contact with an oligonucleotide bound to the helicase template (PDB **1A1V**; Kim et al., 1998) **[18]**; (B) Substitution of tryptophan for arginine at position 501 of NS3, present in patient RF020**.

**Figure 4:**
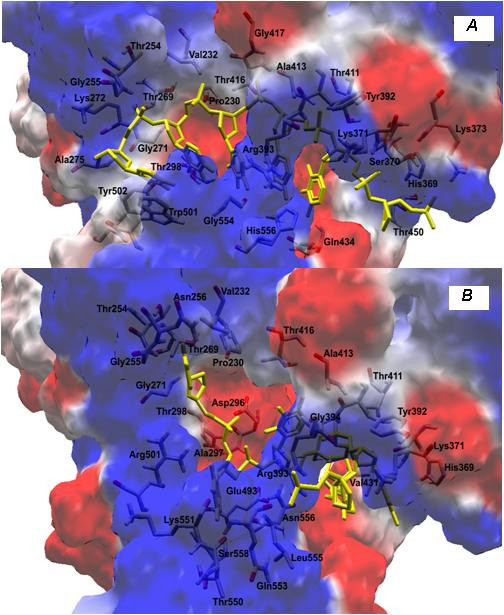
**Docking simulations between helicase and RNA substract: (A) patient RF059 and (B) patient RF020**.

The substitution (W501R; see Table 1) of a non-polar (neutral) amino acid for a polar (positive) residue (Figures 3A and 3B) leads to an increase of positivity in this region and thus alters the interaction of the protein with its environment. Docking simulations between helicase variant and the RNA substract showed that the substitution of tryptophan by arginine at position 501 causes an imbalance in the distribution of loads leading, and forces the RNA to interact with the amino acid, Thr269. In this case, we observed an increase in the positive charges near to R501 (Figures 4A and 4B).

Based on the observation that the oligonucleotide appears to be locked in the binding cleft because a residue in domain 3, Trp501, is stacked against the 3'-terminal base, Kim *et al*. [18], proposed that ATP binding, and the subsequent closure of the cleft between domains 1 and 2, will lead to a ratcheting of Trp501 past 1 or 2 nucleotides. Consequently, the protein would move towards the 5'-end of the bound nucleic acid. After ATP is hydrolyzed and Trp501 is once more locked into a place acting as a bookend, the cleft opens and RNA slides through to the other side of the protein. Consequently the replacement of W501 prevents the correct movement of the protein in the "ratcheting inchworm" model first proposed for HCV helicase by Kim *et al*. [18].

**Figure 5:**
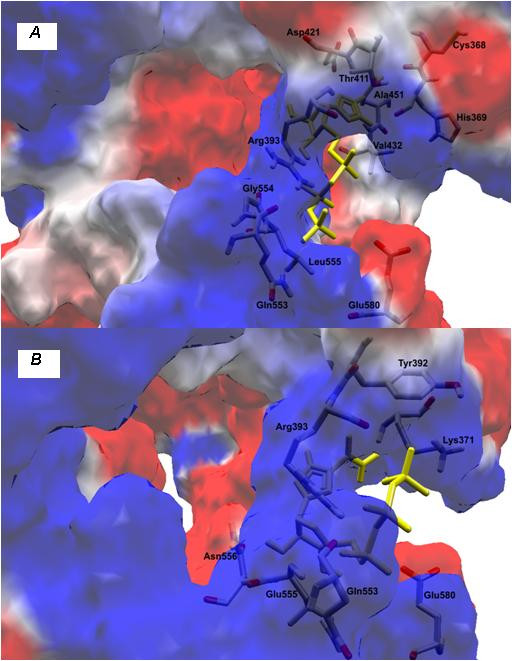
**Docking simulations between helicase and Ribavirin substract: (A) patient RF059 and (B) patient RF020**.

Since interferon-alpha did not act as a protease inhibitor, we also performed docking simulations between the variant protein W501R and the compound Ribavirin to find a correlation between the substitutions found in Helicase and the response of patients to treatment. This inhibitor connects to altered Helicase the same way that as in Helicase wild type, independent of the substitution of tryptophan by arginine at position 501, and thereby maintaining its function (Figures 5A-5B). From this, we infer that the mutation does not prevent the binding of inhibitor, but indicates that this is not the reason for the lack of response to treatment.

## Conclusions

Natural occurrence of amino acids substitution in non-responder HCV patients has been explored for important sites in relation to the perfect excise of helicase function. Correlations of the variants L210E and W501R with patients' responses to the treatment may not be valid at this time because other forms of viruses that have the helicase in their wild types are predominant in the patient, thus ensuring viral replication. It is important to find these variants so that future enzymatic studies can be carried out on them. Individual analysis of treatment with future inhibitors can be important to treatment response.

## Competing interests

The authors declare that they have no competing interests.

## Authors' contributions

PJSP carried out the experiments, acquisition of data, analysis and interpretation of data and drafting the manuscript; HAA and MSP carried out the molecular modeling and suggestions in this manuscript; IMVGCM participated in the study and made suggestions to the manuscript; JRRP and MLN participated in the study; PR conceived the study, participated in its analysis and coordination and suggestions in this manuscript. All authors read and approved the final manuscript.

## Supplementary Material

Additional file 1 Methods, Figure S1 and S2 Click here for file
