# Supplementary material for: Structural studies of Helicase NS3 variants from Hepatitis C virus genotype 3 in virological sustained responder and non-responder patients
Source: BMC Res Notes. 2010 Jul 14;3:196. doi: 10.1186/1756-0500-3-196 (PMC2919562; doi:10.1186/1756-0500-3-196)
Supplement: Additional file 1 — Methods, Figure S1 and S2 [file 1756-0500-3-196-S1.DOC]

**ADDITIONAL FILE 1**

***METHODS***

***RNA Extraction and Reverse Transcription***

A reverse transcription reaction was performed in a total volume of 20.0 µl using a random oligonucleotide primer, and the reactions were performed according to the specifications of the High-Capacity Archive Kit (Applied Biosystems). The mixture was kept at 25°C for 10 minutes and then at 37ºC for 2 hours. Then the material was amplified, using specific primers for the protease and helicase portions of NS3.

***Design of the initializer oligonucleotides***

Oligonucleotide primers were designed to amplify the coding sequence of the helicase domain (181-631 aa). The initializer oligonucleotide primers (forward 5' GGAATTCCATATGTCCCCATCTTTCTCTGACAATTCAACT3' with underlined NdeI site, and reverse 5' CGCGGATCCTCAGGTGGTTACTTCCAGATC 3', with underlined BamHI site) generate amplification products of 1353 bp. The primers were synthesized so as to contain BamHI and NdeI restriction enzyme sites, in order to permit the binding of the amplified fragments in expression vector PET 28A that also has restriction sites for these endonucleases. Internal initializer primers for the complete helicase sequence were also synthesized, to enable their full sequencing (Seq787F 5' GCCAAA CTGACCTATTCCAC 3'; Seq980F 5' AGCATCACTGTGCCACATTC 3'; Seq1216F 5' GTCGTAGTTTGCGCTACTG 3'; Seq1654R 5' GCTTAGTCTGTGACAGAAAGTG 3'; Seq1454R 5' ATT CCAGACGGTCTTTCACC 3'; Seq1005R 5' GTTAGAATGTGGCACAGTGATG 3'; M13 forward 5' GTAAAACGACGGCCAG 3'; M13 reverse 5´ caggaaaCagctatgac 3´).

***cDNA amplification for the helicase domain***

The reactions were performed in a total volume of 20μl containing 100 to 500 ng of cDNA, 1.6x buffer, dNTP mix (200 mM), MgCl^2+^ (4.0 mM), primers (0.5 mM) and Taq DNA polymerase (5 units). In all reactions, one of the tubes received no DNA (contamination control). The cycling comprised an initial step of 2 min at 94°C, 40 cycles of 1 min at 94°C, 1 min for annealing of the primers at a temperature of 60°C to 55°C (for protease and helicase, respectively), 3 min at 72°C for extension of the chains, and finally 15 min at 72°C for final extension. The amplification primers generated products of 540 bp and 1350 bp for protease and helicase, respectively.

***Cloning***

After verifying the amplification of the fragments of interest, they were cloned into cloning vector PCR-XL-TOP. The stages of ligation with the fragment of the plasmid of interest, as well as the transformation into chemically competent bacteria (TOP10) were processed according to the specifications of the Topo XL PCR Cloning Kit (Invitrogen, Life Technologies). The bacteria were grown at 37°C in LB solid culture medium containing 50 µg/ml of the antibiotic kanamycin. To confirm the insertion of the product of interest, after approximately 16 h of growth, the plasmid DNA was amplified using the M13 forward and reverse primers. The reactions were performed in a total volume of 15 μl, containing 1.0 µl culture medium, 1.0x buffer, dNTP mix (125 mM), MgCl^2+^ (1.5 mM), primers (0.14 mM) and Taq DNA polymerase (5 units). In all reactions, there was one test tube that did not receive the culture (contamination control).

Cycling comprised an initial stage of 3 min at 95ºC, 35 cycles of 40 sec at 95ºC, 40 sec for primer annealing at 55ºC, 55 sec at 72ºC for chain extension, and a final extension cycle of 10 min at 72°C. The primers generated products of approximately 150 bp (plasmid) and 1500 bp (helicase + plasmid).

The positive clones were then selected and inoculated in 3.5 ml of LB liquid culture medium containing 50 mg/ml kanamycin. From each patient, five clones of the protease fragment and five clones of the helicase fragment were selected for purification and sequencing. Growth was conducted at 37°C under agitation (250 rpm) for approximately 16 hours. Then, the plasmids were purified from the bacteria culture, using a SNAP Miniprep Kit (Invitrogen, Life Technologies) according to the manufacturers’ instructions.

***Sequencing***

The purified fragments were cloned and sequenced in an ABI Prism 377 automatic sequencer (Applied Biosystems Inc) for confirmation of their sequences. The sequencing reaction was performed in a total volume of 10.0µl containing: Big Dye Terminator (2.0µl), Save Money Buffer (2.5x), primer (1.0 µM), and 2.0 µl of cloned fragment sample. Cycling comprised a first step of 10 sec at 96ºC, 35 cycles of 10 sec at 96ºC, 5 sec for primer annealing at about 50ºC, and 3 min at 60ºC for chain extension. It has been proposed to use five clones of each helicase fragment sample for sequencing.

For the sequencing of the amplification products corresponding to the helicase domain (1353 bp), in addition to the M13 forward and reverse initializers, another three pairs of primers internal to the sequence were necessary, to cover the entire amplification product of 1353 bp. Thus, a total of eight primers were needed for the sequencing reactions of each clone for each sample.

### *Sequence and secondary structure analysis*

An initial assessment to confirm that the sequences obtained from the clones were NS3 was performed using the PSI-BLAST alignment tool [1], [2]. Then, the sequences were analyzed for their quality, using the PHRED / PHRAP / CONSED program found at http://adenina.biomol.unb.br/phph. In addition to assessing the quality of the sequences, the program allows the assembly of consensus sequences. A consensus sequence (contig) for each clone was assembled from the four pairs of oligonucleotide primers.

The NS3 multiple sequence alignment was performed using the CLUSTAL W alignment tool [3] and visualized using the Jalview program [4], to identify possible regions where substitutions occurred. Subsequently, the alignment was manually checked for mismatch. NS3 homologues were identified using the PSI-BLAST [1], [2] search program in the PDB protein database [5]. The prediction of the NS3 secondary structure was made using the Jnet prediction method [6], to identify possible changes in secondary structure because of changes occurring in the clones.

***REFERENCES***

1. [AA](http://www.ncbi.nlm.nih.gov/pubmed?term="Schäffer AA"%5BAuthor%5D), [Aravind L](http://www.ncbi.nlm.nih.gov/pubmed?term="Aravind L"%5BAuthor%5D), [Madden TL](http://www.ncbi.nlm.nih.gov/pubmed?term="Madden TL"%5BAuthor%5D), [Shavirin S](http://www.ncbi.nlm.nih.gov/pubmed?term="Shavirin S"%5BAuthor%5D), [Spouge JL](http://www.ncbi.nlm.nih.gov/pubmed?term="Spouge JL"%5BAuthor%5D), [Wolf YI](http://www.ncbi.nlm.nih.gov/pubmed?term="Wolf YI"%5BAuthor%5D), [Koonin EV](http://www.ncbi.nlm.nih.gov/pubmed?term="Koonin EV"%5BAuthor%5D), [Altschul SF](http://www.ncbi.nlm.nih.gov/pubmed?term="Altschul SF"%5BAuthor%5D). Improving the accuracy of PSI-BLAST protein database searches with composition-based statistics and other refinements. **Nucleic Acids Res** 2001, 29: 2994-3005.
2. [Altschul SF](http://www.ncbi.nlm.nih.gov/pubmed?term="Altschul SF"%5BAuthor%5D), [Madden TL](http://www.ncbi.nlm.nih.gov/pubmed?term="Madden TL"%5BAuthor%5D), [Schäffer AA](http://www.ncbi.nlm.nih.gov/pubmed?term="Schäffer AA"%5BAuthor%5D), [Zhang J](http://www.ncbi.nlm.nih.gov/pubmed?term="Zhang J"%5BAuthor%5D), [Zhang Z](http://www.ncbi.nlm.nih.gov/pubmed?term="Zhang Z"%5BAuthor%5D), [Miller W](http://www.ncbi.nlm.nih.gov/pubmed?term="Miller W"%5BAuthor%5D), [Lipman DJ](http://www.ncbi.nlm.nih.gov/pubmed?term="Lipman DJ"%5BAuthor%5D). Gapped BLAST and PSI-BLAST: a new generation of protein database search programs. **Nucleic Acids Res** 1997, 25: 3389-3402.
3. Thompson JD, Higgins DG, Gibson TJ. CLUSTAL W: improving the sensitivity of progressive multiple sequence alignment through sequence weighting, position-specific gap penalties and weight matrix choice. **Nucleic Acids Res** 1994, 22: 4673-4680.
4. Clamp M, Cuff J, Searle SM, Barton GJ. The Jalview Java alignment editor. **Bioinformatics** 2004, 20: 426-427.
5. Berman HM, Bhat TN, Bourne PE, Feng Z, Gilliland G, Weissig H, Westbrook J. The Protein Data Bank and the challenge of structural genomics. **Nat Struct Biol** 2000, **7** Suppl, 957-959.
6. Cuff JA, Barton GJ. Application of multiple sequence alignment profiles to improve protein secondary structure prediction. **Proteins** 2000, 40: 502-511.

**FIGURES**

**
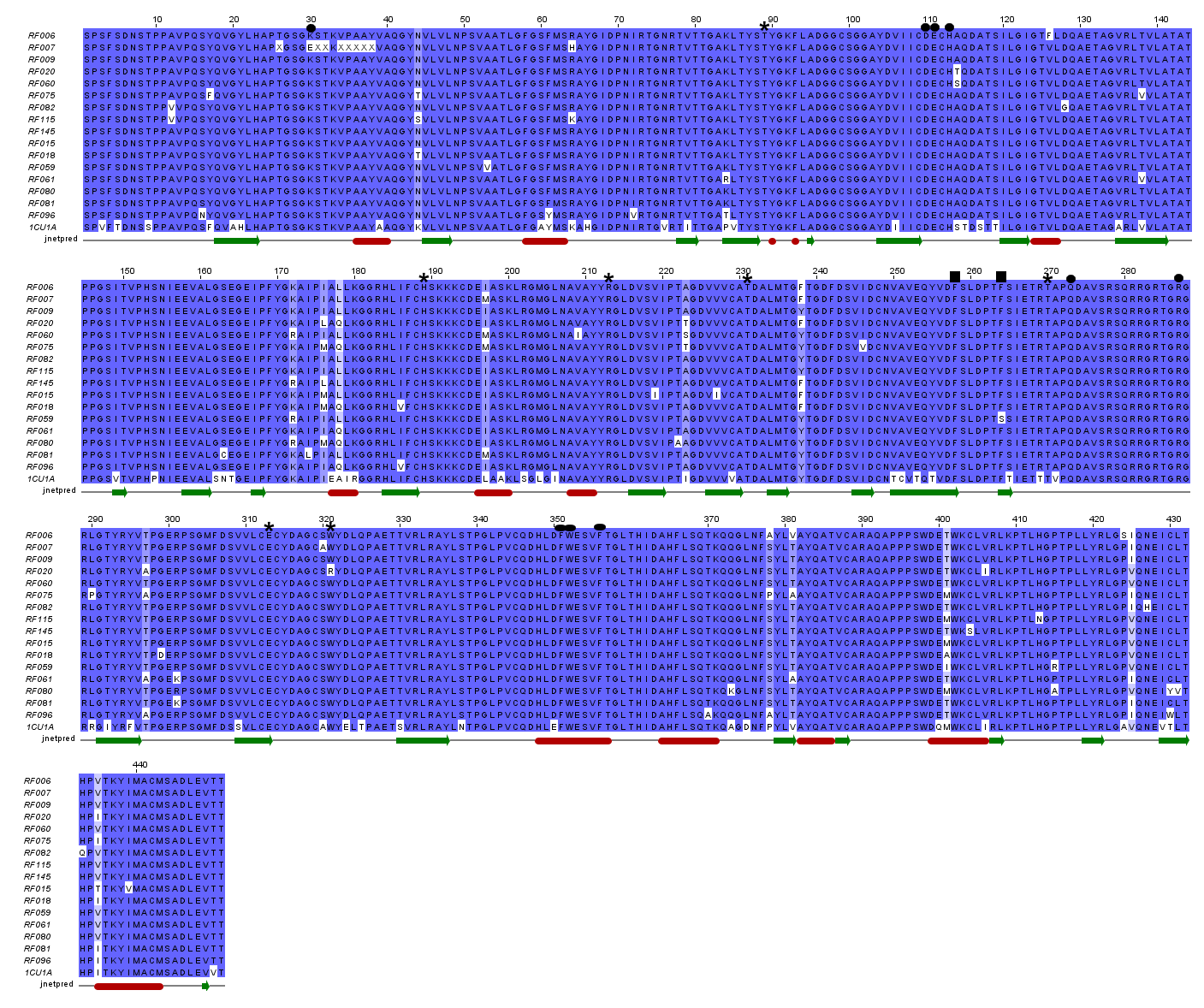
**

**Figure S1:** Multiple sequence alignment of NS3 helicase showing the substitutions found. Identical residues are indicated in darker blue and non-identical residues in lighter blue. The symbols represent: • ATP-binding site, * RNA binding site, ■ phenylalanine loop, and the tip of the loop (hydrophobic region). The positions of the residues corresponding to the coding sequence of the helicase domain and not to NS3 which must be added additional 180 residues. For secondary structure prediction, the helices are indicated in red, the strands in green, and the random regions in black.

**
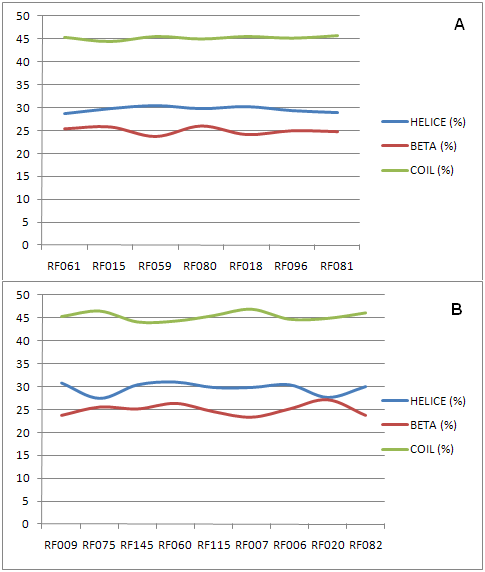
**

**Diagram I**: The PROTEUS analysis showing the percentage of helice, beta sheet and coil in the helicase protein. (A) Virological sustained response patients. (B) Non-responder patients.


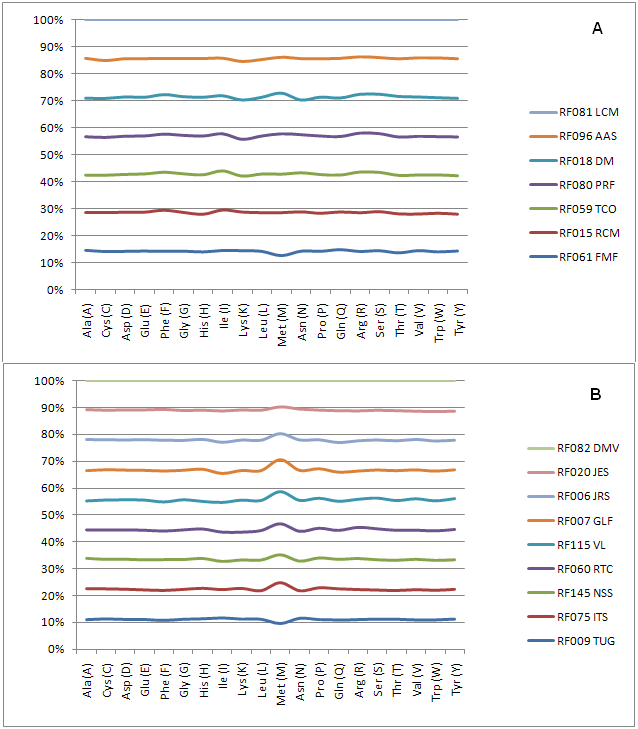


**Diagram II**: The PROTPARAM analysis showing the change of aminoacids composition in the helicase protein. (A) Virological sustained response patients. (B) Non-responder patients.


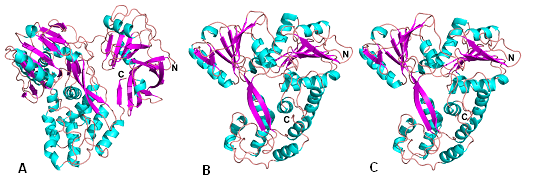


**Figure S2:** Diagram of the tertiary structure: (A) 1CU1 (template), (B) patient RF059, (C) patient RF082. The helices are indicated in light green, the strands in light pink, and the coil in dark pink.
